# Supplementary material for: Content-rich biological network constructed by mining PubMed abstracts
Source: BMC Bioinformatics. 2004 Oct 8;5:147. doi: 10.1186/1471-2105-5-147 (PMC528731; doi:10.1186/1471-2105-5-147)
Supplement: Additional File 2 — The original results of the above study (non-essential files are deleted to keep the file size under the limit set by BMC bioinformatics). [file 1471-2105-5-147-S2.bz2 › chilibotAdditionalFile2/dip05/376ID10713102E9789/html/NGFB_NTRK1.html]

 


 **NGFB** and **NTRK1** 
  
Found 1439 abstracts in PubMed, retrieved 05.  
 

 What does Google say? 
 PDF only 
| .edu only 

---

**Interactive relationship** (e.g. stimulation, inhibition, etc)

**Non-interactive relationship** (e.g. studied together, co-existance, homology, etc.)

- In particular, the putative role of NGF  [ **NGFB** ]  and its receptor TrkA  [ **NTRK1** ]  in immune system homeostasis has become a much studied topic.  Ref: 12892403 J Anat, 2003
- However, NGF  [ **NGFB** ]  rescue from growth factor withdrawal requires NGF  [ **NGFB** ]  signalling through the high affinity tyrosine kinase receptor  [ **NTRK1** ]  TrkA  [ **NTRK1** ] , while NGF  [ **NGFB** ]  rescue from TNFalpha promoted cell death requires NGF  [ **NGFB** ]  signalling through the low affinity p75NTR receptor.  Ref: 12882321 Aging Cell, 2003
- Western blot analysis confirmed these results, showing that the lacrimal gland expresses NGF  [ **NGFB** ] , BDNF, NT3, and NT4 as well as the NTrs TrkA  [ **NTRK1** ] , TrkB, and TrkC and the p75 protein.  Ref: 12882781 Invest Ophthalmol Vis Sci, 2003
- RT PCR showed positive bands at the appropriate sizes for NGF  [ **NGFB** ] , BDNF, NT3, and NT4, and for the receptors TrkA  [ **NTRK1** ] , TrkB, TrkC, and p75.  Ref: 12882781 Invest Ophthalmol Vis Sci, 2003
- To determine which of the neurotrophins NTs nerve growth factor  [ **NGFB** ]  NGF  [ **NGFB** ] , brain derived neurotrophic factor BDNF, neurotrophin 3 NT3, and neurotrophin 4 5 NT4 and their receptors NTrs, TrkA  [ **NTRK1** ] , TrkB, TrkC, and p75, are present in the adult rat lacrimal gland.  Ref: 12882781 Invest Ophthalmol Vis Sci, 2003
- Quantitative in situ hybridization was used to detect changes in neuronal PACAP mRNA expression and to correlate this expression with neuronal mRNA expression of the NGF  [ **NGFB** ]  receptor tyrosine kinase trk A  [ **NTRK1** ] .  Ref: 12890505 Neuroscience, 2003
